# Reproducibility of identical solid phantoms

**DOI:** 10.1117/1.JBO.27.7.074713

**Published:** 2022-02-02

**Authors:** Fangzhou Zhao, Pietro Levoni, Lorenzo Frabasile, Hong Qi, Michele Lacerenza, Pranav Lanka, Alessandro Torricelli, Antonio Pifferi, Rinaldo Cubeddu, Lorenzo Spinelli

**Affiliations:** a Politecnico di Milano, Dipartimento di Fisica, Milano, Italy; b Harbin Institute of Technology, School of Energy Science and Engineering, Harbin, China; c Instituto di Fotonica e Nanotecnologie (IFN), Consiglio Nazionale delle Ricerche (CNR), Milano, Italy

**Keywords:** tissue-like solid phantoms, phantom reproducibility, instrument comparison, time-resolved diffuse optics

## Abstract

**Significance:**

Tissue-like solid phantoms with identical optical properties, known within tolerant uncertainty, are of crucial importance in diffuse optics for instrumentation assessment, interlaboratory comparison studies, industrial standards, and multicentric clinical trials.

**Aim:**

The reproducibility in fabrication of homogeneous solid phantoms is focused based on spectra measurements by instrument comparisons grounded on the time-resolved diffuse optics.

**Approach:**

Epoxy-resin and silicone phantoms are considered as matrices and both employ three different instruments for time-resolved diffuse spectroscopy within the spectral range of 540 to 1100 nm. In particular, we fabricated two batches of five phantoms each in epoxy resin and silicone. Then, we evaluated the intra- and interbatch variability with respect to the instrument precision, by considering the coefficient of variation (CV) of absorption and reduced scattering coefficients.

**Results:**

We observed a similar precision for the three instruments, within 2% for repeated measurements on the same phantom. For epoxy-resin phantoms, the intra- and the interbatch variability reached the instrument precision limit, demonstrating a very good phantom reproducibility. For the silicone phantoms, we observed larger values for intra- and interbatch variability. In particular, at worst, for reduced scattering coefficient interbatch CV was about 5%.

**Conclusions:**

Results suggest that the fabrication of solid phantoms, especially considering epoxy-resin matrix, is highly reproducible, even if they come from different batch fabrications and are measured using different instruments.

## 1 Introduction

The possibility to probe human tissue in depth and non-invasively by visible and near-infrared light^1^^,^^2^ pushed scientists and technologists to develop physics, methodologies, and instrumentation to study photon propagation in highly scattering media. In the 600 to 1100 nm wavelength range, human tissues are diffusive media characterized by a scattering coefficient much higher than the absorption. Furthermore, visible and near-infrared diffuse spectroscopy (NIRS) experienced a huge improvement as a suitable technique for detecting the structural or functional information inside the tissues by studying the light-tissue interaction process.

From the first pioneering basic studies on photon migration^3^^–^^6^ to the more recent NIRS instrumentation involved in clinical trials,^7^^–^^12^ it was clear that the availability of phantoms mimicking the optical properties and also the structures of biological tissues was required for reliability of the results attained. Whatever approach is exploited for NIRS—continuous wave, time domain, or frequency domain; the geometry adopted—transmittance or reflectance; the reconstruction modality utilized—tomography or topography; the instrument configuration employed—a multichannel imaging system or a single-channel monitor; the clinical application aimed for—brain, breast, or muscle, diffuse samples with reliable and invariable properties are fundamental and important tools for (1) the development of novel approaches in basic research; (2) the instrumentation assessment in laboratory environment^13^^,^^14^; (3) interlaboratory comparison studies^15^^–^^17^; and (4) quality check in clinics.^18^

In this framework, the possibility to have phantoms with optical properties known with few percent of uncertainty is highly advisable. This is the pivotal problem for accuracy. Though it has been fundamentally solved for liquid phantoms,^19^ it remains an open issue for solid phantoms, where an accurate estimation of the optical properties is not straightforward to obtain, neither *a priori*—starting from properties of raw materials—nor *a posteriori*—from a direct measurement on diffusive phantoms.

Since the use of solid phantoms has obvious advantages for practical reasons and as an accurate estimation of their optical properties seems for now too far, we can focus on another aspect of equal importance that should characterize diffuse phantoms: their reproducibility. If a good level of reproducibility is guaranteed in phantom fabrication to yield a set of identical phantoms, we can accomplish many important issues toward a more standardized development of NIRS instruments. For example, these phantoms could be equally useful in interlaboratory studies or in multicentric clinical trials, even if the accurate values of their optical properties are not known, at least they are guaranteed with known uncertainty.

The goal of this paper is to assess the degree of attainable reproducibility during solid phantom fabrication in terms of the absorption coefficient $\mu_{a}$ and the reduced scattering coefficient $\mu_{s^{\prime }}$. We considered phantoms obtained both from the same preparation and different preparations. Moreover, we considered two different materials commonly exploited as matrix for phantom realization: epoxy resin and silicone. Optical properties of the phantoms were assessed by means of three different systems for time-domain NIRS.

After the detailed description of the phantom fabrication in Sec. 2.1, the instruments used for their characterization and the data analysis methods are reported in Sec. 2.2. Then, the results and discussion are presented in Sec. 3. Finally, some conclusions are drawn in Sec. 4.

## 2 Materials and Methods

### 2.1 Phantom Preparation

#### 2.1.1 Epoxy resin

Epoxytable10 (part A 100 and part B 25, Resinpro, Italy) was the epoxy resin used to prepare the phantoms. This resin can tolerate sample thickness up to 10 cm without adverse thermal effects during the curing time. The absorption properties were obtained by first preparing a concentrated stock solution made by mixing 120 mg of toner (Infotec toner black 46/l) to 100 g of part A. A proper amount of this stock solution was added to the part A for the required absorption value. Titanium oxide powder was used to provide the scattering properties. Each phantom was prepared to obtain a cylindrical solid phantom of 63 mm in diameter and 40 mm in height. The liquid resin was poured in a cylindrical Nalgene container (Sigma Aldrich). When solid, the resin does not adhere to this plastic.

To reach the nominal values at 690 nm for the reduced scattering coefficient of $10\;{\mathrm{cm}}^{-1}$ and for the absorption coefficient of $0.1\;{\mathrm{cm}}^{-1}$, 156.5 mg of ${\mathrm{TiO}}_{2}$ powder and 1.64 g of stock solution were mixed to 110.4 g of part A. The solution was stirred by a mixer (Ergo Mix 450W, Bosch) for 3 min and homogenized for 3 min (OV5, Velp Scientifica). Then 28.0 g of part B was added and the whole mixture was stirred for 2 min and homogenized for 2 min. ${\mathrm{TiO}}_{2}$ powder and stock solution were weighted by a precision balance (res. 0.01 mg) while for parts A and B a normal balance (res. 0.01 g) was used. After this process, the resin was set in a plastic bell jar connected to a vacuum pump (XDS5, Edwards) to remove the air bubbles. The solution was then left at room temperature for curing. This procedure was repeated from scratch for each phantom. Five phantoms were prepared in the same day, defined as batch 1, and five a few days later, defined as batch 2.

#### 2.1.2 Silicone

Silicone Elastomer Sylgard 184 (Base 10 and Curing Agent 1, Dow Corning) was used to prepare the silicone phantoms. The size of these phantoms was the same of the epoxy-resin ones cited earlier. Scattering and absorption properties were obtained with a similar procedure using ${\mathrm{TiO}}_{2}$ powder and a stock solution of 120-mg toner in 100-g Base. For the nominal values at 690 nm of $10\;{\mathrm{cm}}^{-1}$ reduced scattering and $0.1\;{\mathrm{cm}}^{-1}$ absorption, 127.6 mg of ${\mathrm{TiO}}_{2}$ and 6.4 g of stock solution were added to 121.6 g of Base. As compared to the resin, the different amount of stock solution is related to a different dispersion of the toner in the silicone. The mixture was stirred for 3 min and homogenized for 2 min. The addition of 12.8 g of the curing agent completed the recipe. Again, following 2-min stirring and 1-min homogenizing, air bubbles were removed from the solution by the vacuum pump system. The curing procedure was obtained by keeping the samples in the oven (UT 20P, Heraeus) at 65°C overnight. As for the silicone, five phantoms were prepared in the same day, defined as batch 1, and five a few days later, defined as batch 2.

### 2.2 Experimental Setups

#### 2.2.1 System 1: broadband diffuse optical spectroscopy

A broadband time-domain diffuse optical spectroscopy device (Diffuse Optical Spectroscopy Laboratory, Department of Physics, Politecnico di Milano, Milan, Italy) capable of measuring the optical property spectra (absorption and reduced scattering) over a wide range of wavelengths was chosen for this study.

**Fig. 1:**
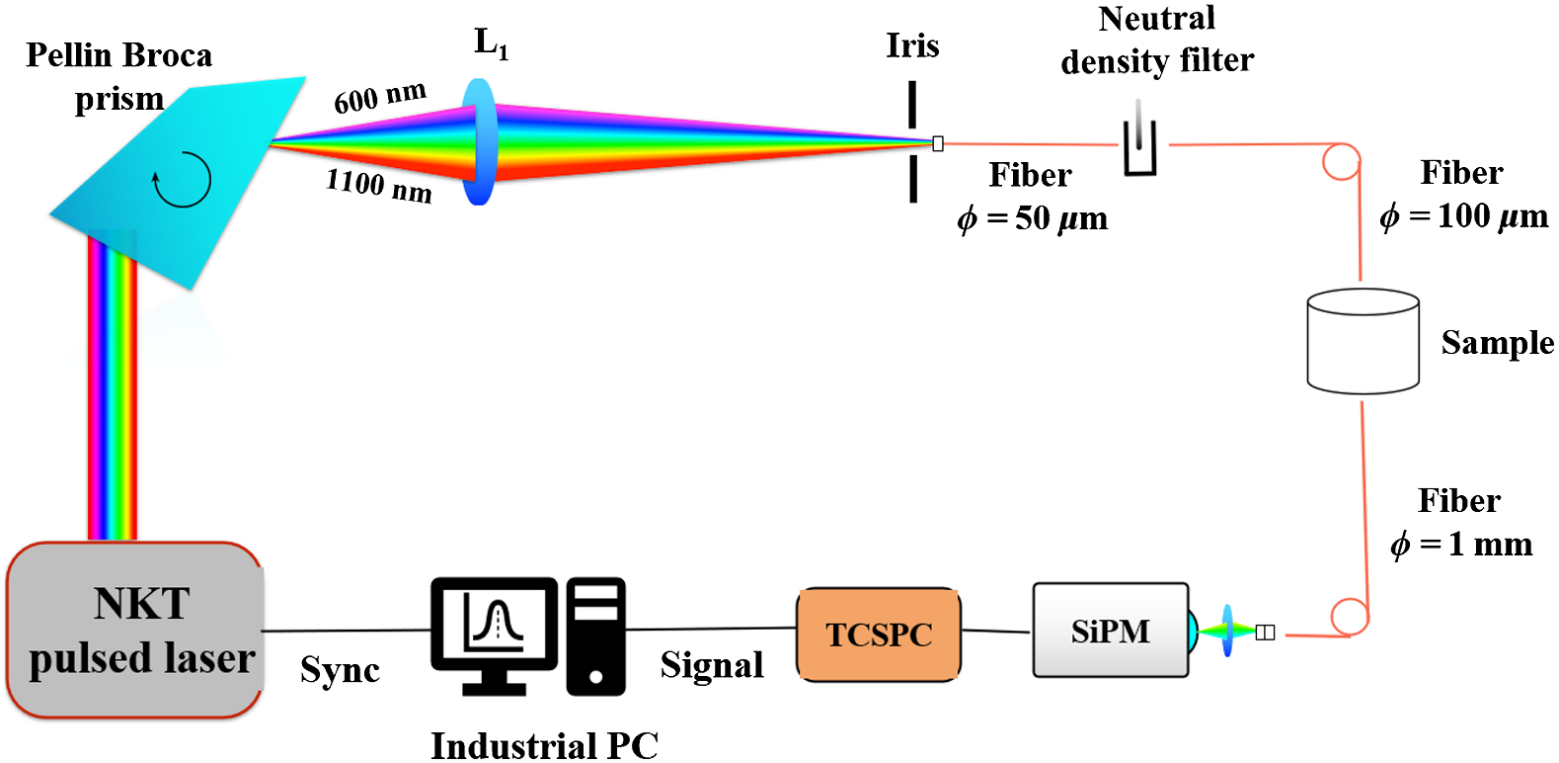
Schematic of the DOS instrument.

The spectral acquisition is achieved by sequentially scanning the sample for the time-of-flight curves over a broad wavelength range (600 to 1100 nm at steps of 10 nm). A supercontinuum white light laser source (SuperK Extreme, NKT Photonics, Denmark) emitting picosecond pulses in the wavelength range 400 to 2400 nm is used as the source. The source is spectrally dispersed using a Pellin Broca prism (B. Halle Nachfl., Germany). The source then passes through a narrow iris and is coupled into a small core (50  μm) graded index fiber. Together these two components restrict the spectral bandwidth of the coupled light within the range of 3 to 9 nm over the wavelength range of interest. Wavelength selection is achieved then by rotating the prism. The laser is operated at a repetition rate of 40 MHz. A variable neutral density filter (Edmund Optics, United States) placed between the source fiber and the sample was utilized to attenuate the source in order to maintain the detection count rate around $5\cdot 10^{5}$ counts per second (roughly 1% of the repetition rate of the laser). The average output power at the sample is about 4 mW per each wavelength. Light diffusively reflected through the sample is collected through a fiber (core diameter 1 mm, 1.5 m in length, step index) and delivered to a Silicon Photomultiplier (SiPM) (S10362-11-050C, Hamamatsu, Japan) detector.^20^ A time-correlated single photon counting (TCSPC) board (SPC-130, Becker & Hickl, Germany) is then used to acquire and analyze the time-resolved signals. The dark noise of the instrument is $2\cdot 10^{4}$ counts per second with an average full width at half maximum (FWHM) of the instrument response function (IRF) of 85 ps over the wavelength range. The schematic of the DOS instrument is shown in Fig. 1.

#### 2.2.2 System 2: PHOOD

The PHOOD device (Photonics for Food Laboratory, Department of Physics, Politecnico di Milano, Milan, Italy) is a multiwavelength, time-domain NIRS device. It is based on an SC450-6W high-power supercontinuum fiber laser (Fianium Ltd., Southampton, United Kingdom) emitting white-light picosecond pulses within the wavelength range 489 to 1800 nm. The laser is characterized by pulse duration of 4 ps, repetition frequency of 40 MHz and a maximum output power of 6 W with average output power density of about 1  mW/nm. Two coupled continuously variable neutral-density filters with optical density 4.0 (Edmund Optics Inc., Barrington, New Jersey, United States) are responsible for adjusting light attenuation.

The injection wavelength is finely selected by a set of 14 band-pass interference filters: a 671-nm MaxLine laser-line filter (Semrock Inc., Rochester, New York, United States), with 2.6-nm bandwidth and optical density >5.0, and 13 TECHSPEC band-pass filters (Edmund Optics Inc., Barrington, New Jersey, United States), with 10-nm bandwidth and optical density >4.0, respectively centered at 540, 580, 610, 632, 650, 690, 730, 780, 830, 880, 940, 980, and 1064 nm. The injection fiber is an FG200LEA step-index multimode fiber (Thorlabs Inc., Newton, New Jersey, United States) with core diameter 200  μm, while the collection fiber is an OM-Giga graded-index plastic optical fiber (FiberFin Inc., Yorkville, Illinois, United States) with core diameter 1 mm.

The detection wavelength, which must be identical to the injection wavelength, is finely selected by a set of 14 band-pass interference filters identical and paired to those in the injection optical system. Single-photon detection is provided by a SiPM module^20^ based on a SiPM detector with active area $1.69\;{\mathrm{mm}}^{2}$ and time resolution of 75 ps.

**Fig. 2:**
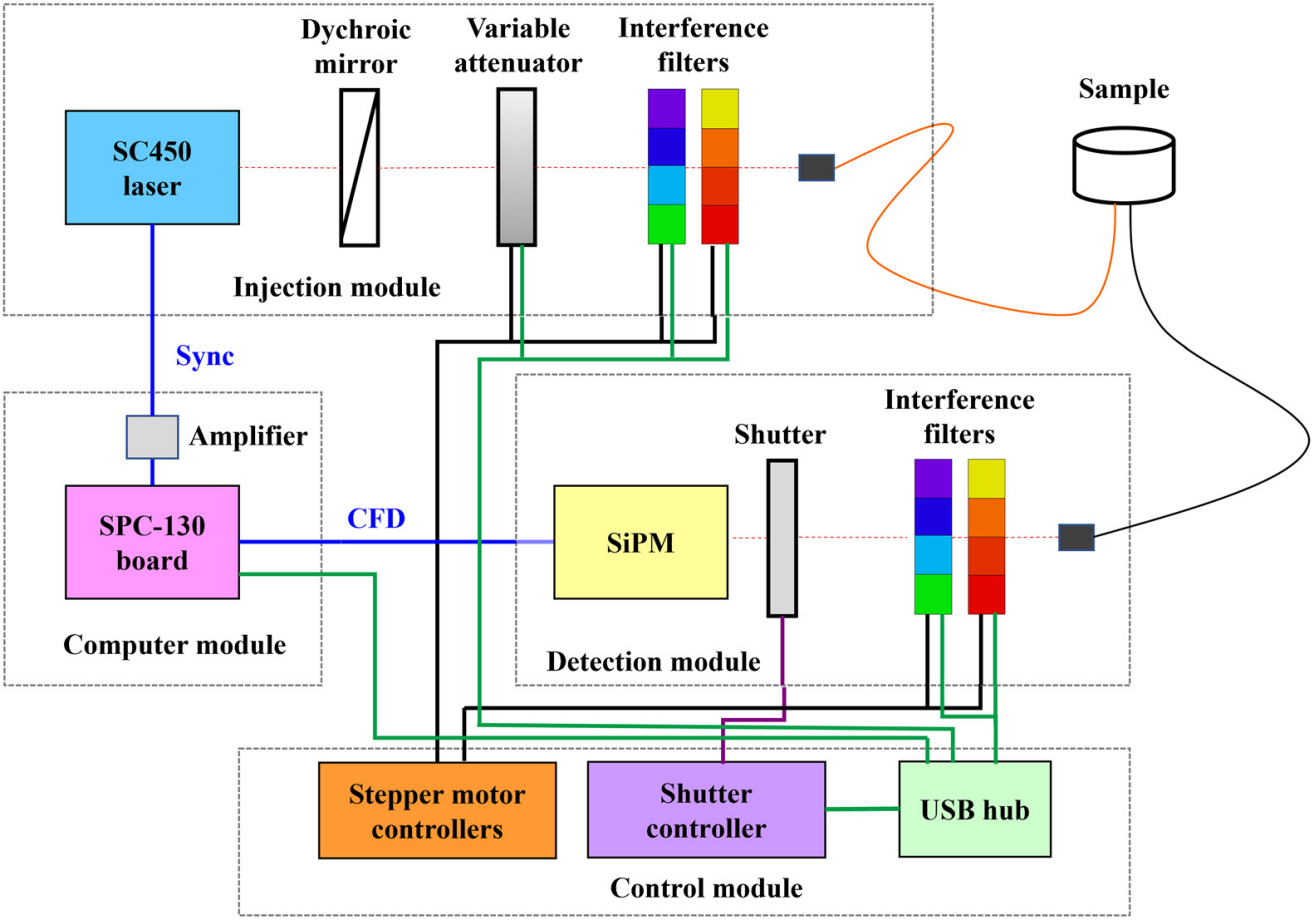
Schematic of the PHOOD instrument.

The data acquisition is performed by an SPC-130 board (Becker & Hickl GmbH, Berlin, Germany) that exploits the TCSPC technique. The FWHM of the IRF is about 100 ps. The background noise accounts for $2\cdot 10^{4}$ counts per second. The measurements were performed setting an acquisition time of 1 s and a counting rate of $5\cdot 10^{5}$ photons per second. The schematic of the PHOOD instrument is shown in Fig. 2.

#### 2.2.3 System 3: NIRSBOX

**Fig. 3:**
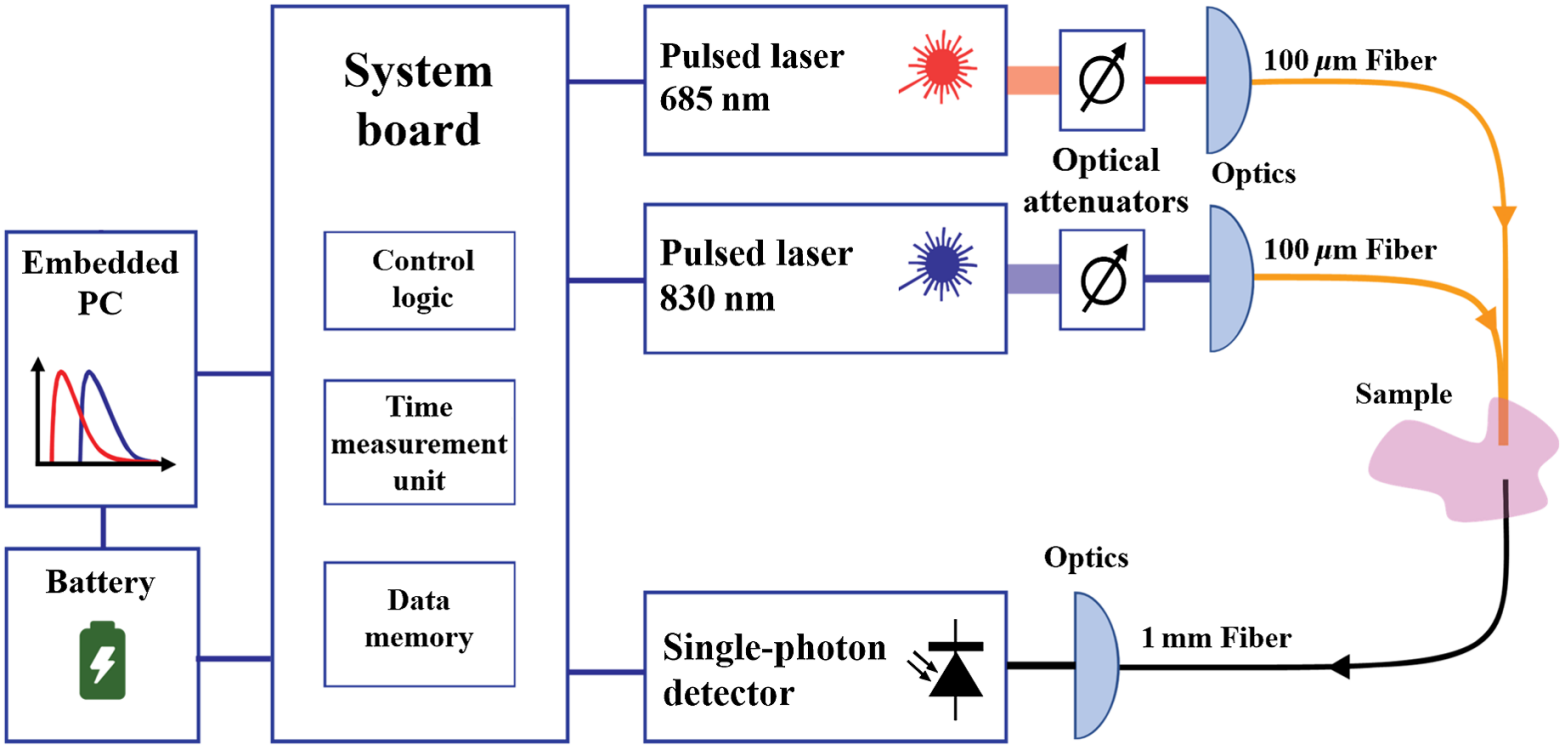
Schematic of the NIRSBOX instrument.

The NIRSBOX device (PIONIRS s.r.l.,^21^ Milan, Italy) is a single-channel, dual-wavelength, TD-NIRS device. It has a compact layout, low energy consumption and it can be battery operated. The two wavelengths, 685 and 830 nm, are generated by two pulsed laser diodes running at a repetition frequency of 53 MHz. The average output power is around 5 mW. Laser light is transferred/collected to/from the phantom through an optical probe (S1-FC, PIONIRS s.r.l., Milan, Italy). The probe is composed by a 1.5-m-long fiber bundle composed by two fibers (100-μm core, multimode graded index, silica) on the injection side and a 1.5-m-long collection fiber (1-mm core, multimode, graded index, POF) both connected to a 90-deg bending optical interface (S1-FC, PIONIRS s.r.l., Milan, Italy). Photons are collected by single-photon detection module, SiPM with an active area of $1.7\;{\mathrm{mm}}^{2}$. The NIRSbox device, through the TCSPC technique, is able to measure the photon distribution of time-of-flight in the sample with a temporal resolution up to 10 ps. The background noise counts ($2\cdot 10^{4}$ counts per second) are kept 2 to 3 orders of magnitude below the peak intensity by shielding the probe with a black fabric. To guarantee a good signal-to-noise ratio during the measurements, the photon count rate is kept around $10^{6}$ counts per second. The integration time was fixed to 500 ms. The IRF has a FWHM <200  ps for both lasers. The schematic of the NIRSBOX instrument is shown in Fig. 3.

### 2.3 Data Analysis

To retrieve the optical properties of different phantoms, i.e., the absorption coefficient $\mu_{a}$ and the reduced scattering coefficient $\mu_{S^{\prime }}$, a model for photon diffusion in turbid homogeneous semi-infinite media was exploited.^22^ In particular, a home-made optimization software, based on Levenberg–Marquardt nonlinear least-squares algorithm, was used for adapting the solution of the time-resolved diffusion equation, obtained considering extrapolated boundary conditions,^22^ to the measured curves, after convolution with the corresponding IRF. During the fitting process, $\mu_{a}$ and $\mu_{S^{\prime }}$ have been assumed as free parameters, while the time origin was fixed. Subtraction of a constant background has been performed if needed, while the fitting range was adapted to each curve, by considering the limits of 50% and 1% of the peak intensity, on the leading and trailing edge, respectively.

### 2.4 Protocols

Phantom measurements were performed in reflectance geometry. For each phantom, the measurements were performed on five different positions. For the measurement on the first position, the injection and the detection points were positioned symmetrically with respect to the phantom center. Then, the phantom was rotated 60 deg for four times, obtaining the five measurements in different positions. The same measurement procedure was repeated on all the 20 phantoms for the three different instruments. In addition, three different interfiber distances were employed using the three instruments to achieve a better representation of the homogenization for the entire phantom. In detail, we chose the interfiber distance of 1.5 cm for the PHOOD device, 2 cm for the DOS device, and 3 cm for the NIRSBOX device during the measurements.

As for the spectral characteristics, measurements were performed at different wavelengths for the three instruments, as follows: with DOS system we considered 26 wavelengths equally spaced in the range of 600 to 1100 nm; with NIRSBOX and PHOOD systems all the available wavelengths were used.

For each phantom, mean values and standard deviations of the optical properties were calculated employing the five repeated measurements on the same phantom. Then, the coefficient of variation (CV), representing the instrument precision, was calculated as the ratio between the pooled standard deviation σ(x) and the pooled average ⟨x⟩ of the 10 phantoms of the same material as follows:

$$\mathrm{CV}=\frac{\sigma (x)}{\left\langle x\right\rangle },$$ (1)

where x is the measured variable, in our case $\mu_{a}$ and $\mu_{S^{\prime }}$. The instrument precision is important for assessing the homogeneity and measurement robustness of the optical properties of a phantom.

As for the phantom comparison within the same batch, the intrabatch CV is adopted to compare the optical properties of the phantoms as follows. First, the mean value of the optical properties for each phantom is calculated as described earlier. Then, from the five mean values of a specific batch the mean value and the standard deviation can be obtained. Finally, the intrabatch CV, adopted to assess the phantom differences within the batch, was calculated as the ratio between the pooled standard deviation and the pooled average of the two batches of the same material.

The interbatch CV is employed to compare batch difference of the same material. For a specific batch, the mean value of the optical properties is obtained by averaging the mean values obtained for each phantom of the batch. Afterward, the CV is calculated from the mean values of the two batches of the same material. Consequently, the interbatch CV can assess the batch quality and compare batch differences.

## 3 Results and Discussion

### 3.1 Optical Properties

**Fig. 4:**
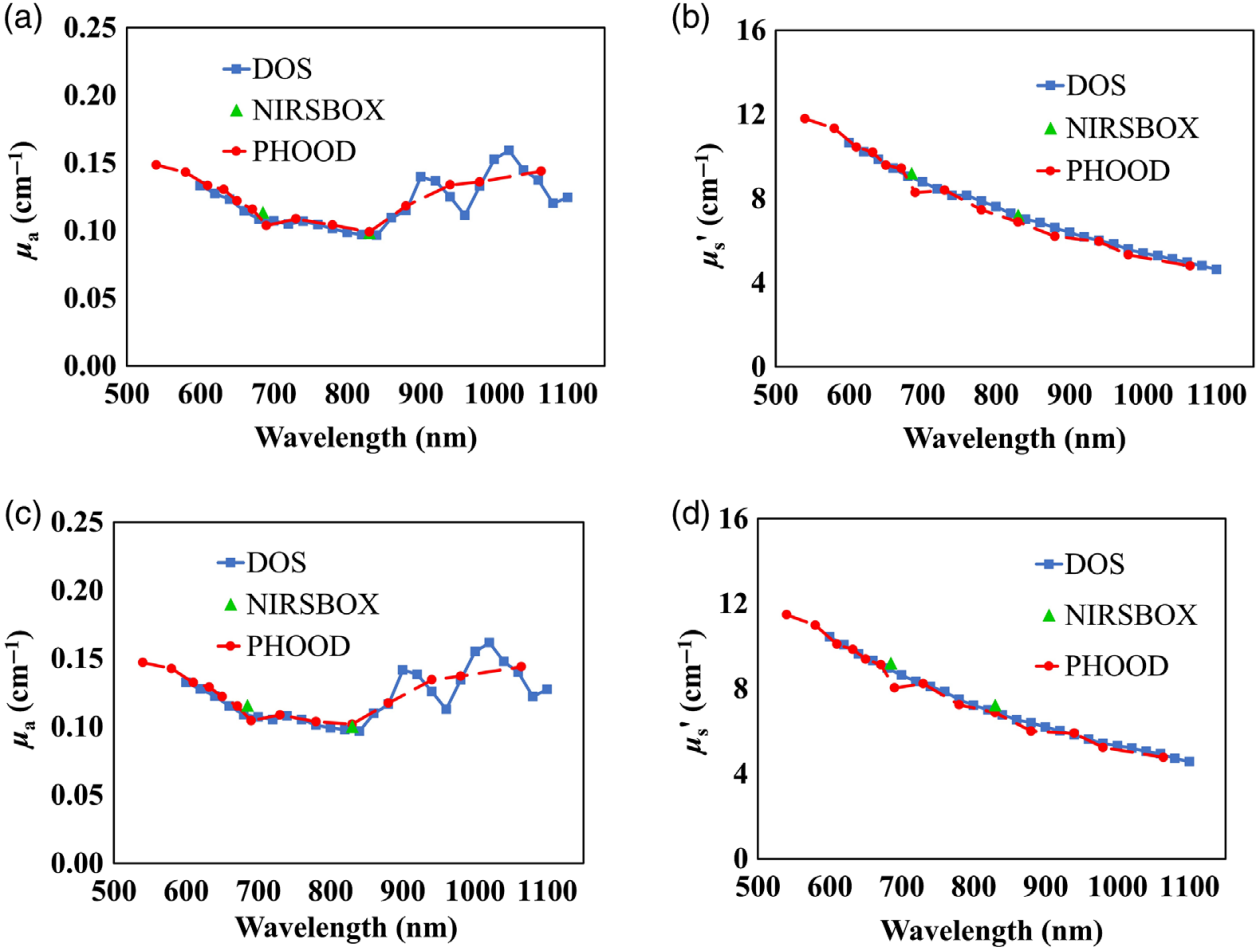
Mean absorption coefficient, panels (a) and (c) and reduced scattering coefficient, panels (b) and (d), as a function of wavelength for phantom 1 of the two epoxy-resin batches. Results obtained employing the three measurement systems are shown.

**Fig. 5:**
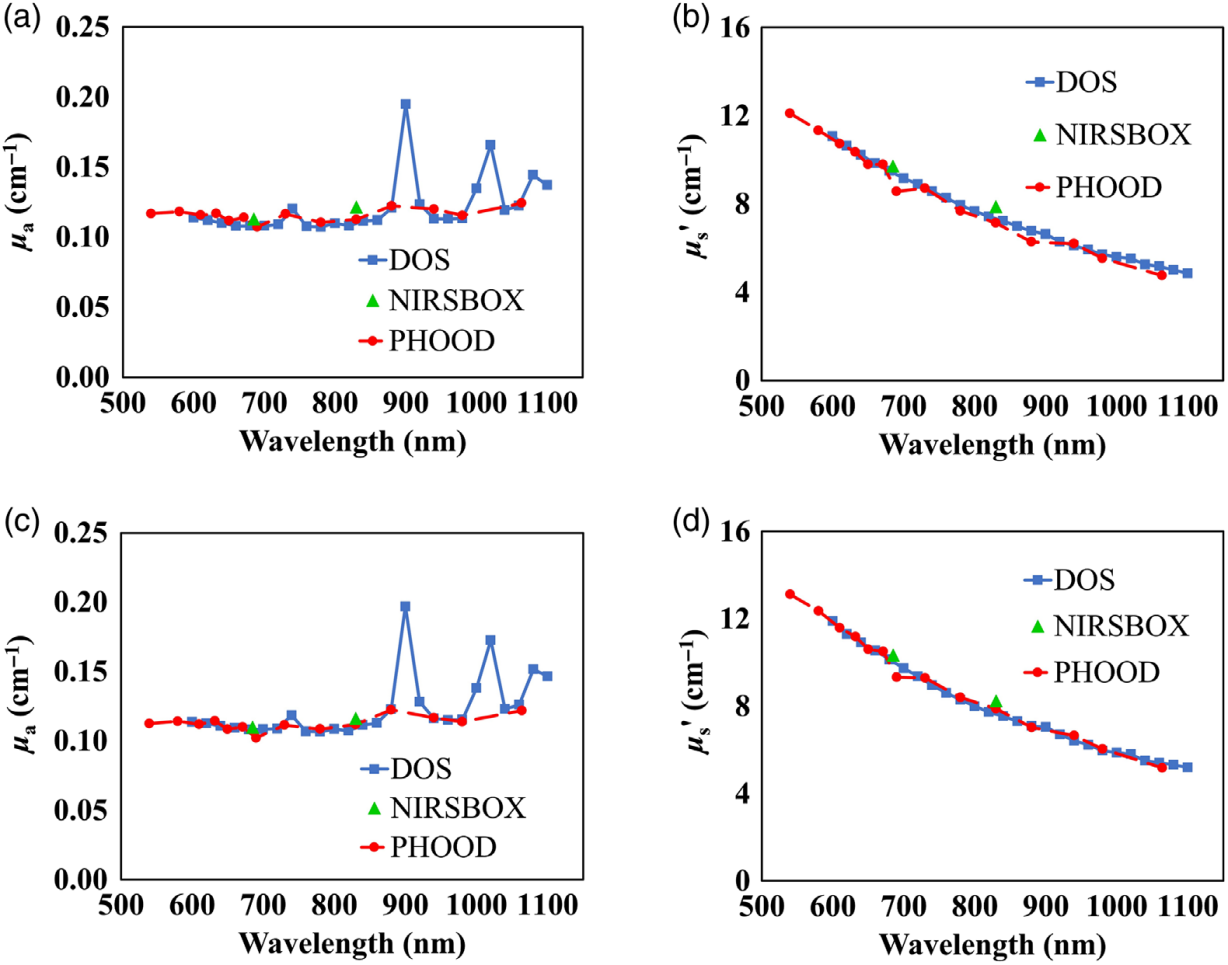
Mean absorption coefficient, panels (a) and (c) and reduced scattering coefficient, panels (b) and (d), as a function of wavelength for phantom 1 of the two silicone batches. Results obtained employing the three measurement systems are shown.

For the five phantoms in a batch, the phantoms are numbered as phantom 1, 2, 3, 4, and 5 to distinguish the difference of the phantoms. The mean absorption and reduced scattering spectra of phantom 1 from the two batches for the epoxy-resin and the silicone material are shown in Figs. 4 and 5, respectively. Here, mean values for phantom 1 are presented as an example of all batches of the two materials. It can be seen that the absorption and reduced scattering spectra measured by the three systems are highly consistent.

The spectra characteristic is continuous with respect to the same instrument. The DOS system exhibits better spectral regions of significant variations in the absorption because of the higher number of wavelengths adopted. In particular, for the epoxy-resin phantom, in Figs. 4(a) and 4(c), two significant absorption peaks are visible around 900 and 1020 nm. For the silicone phantom, in Figs. 5(a) and 5(c), three significant absorption peaks are observed around 900, 1020, and 1100 nm. These different spectral characteristics of the materials are ascribed to the resin and silicone matrix, respectively. With regards to reduced scattering spectra, no distinctive features are observed apart from the expected power law dependence with wavelength for both materials.

### 3.2 Instrument Precision

**Fig. 6:**
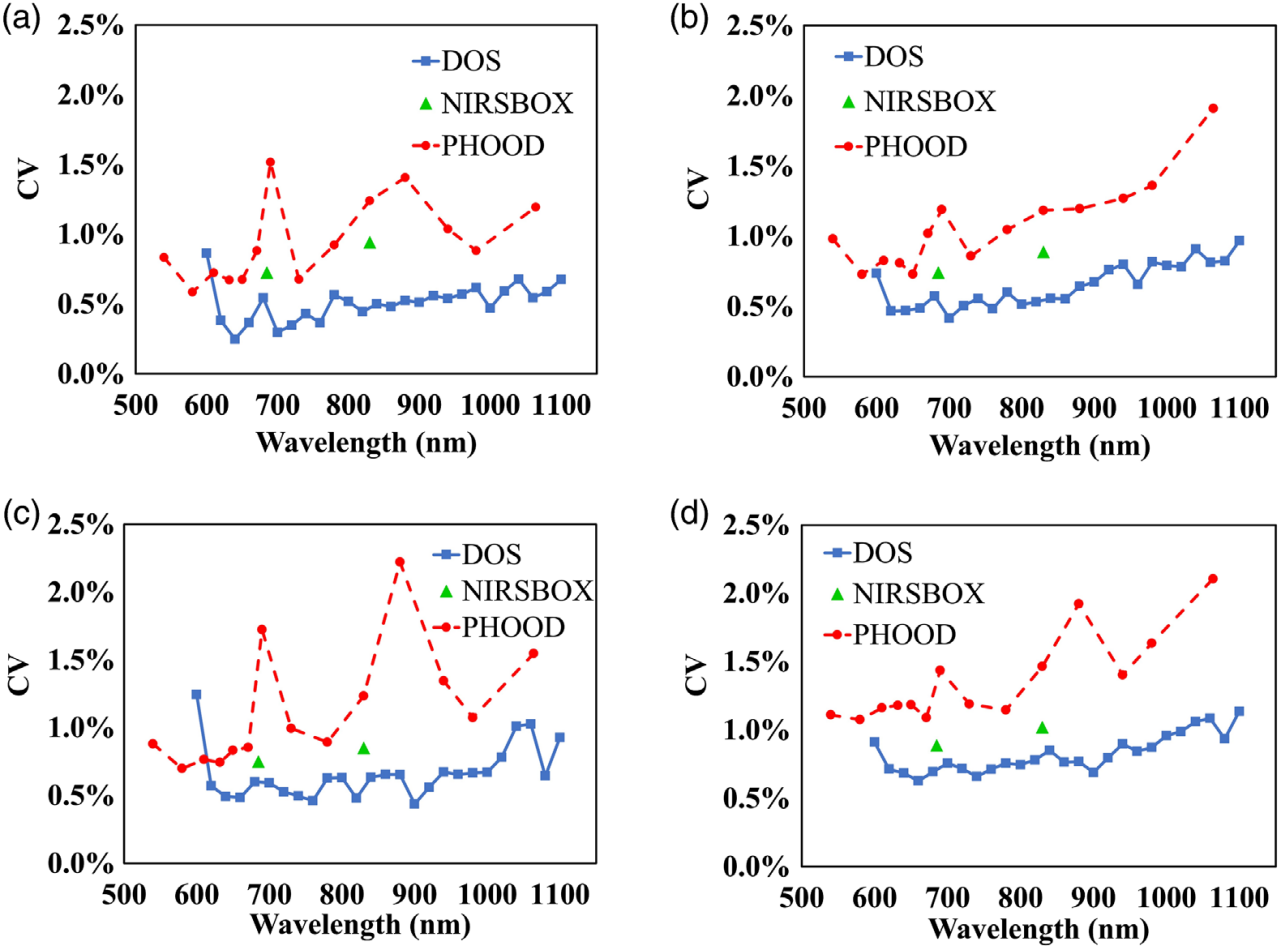
Precision of the three instruments as a function of the wavelength, for absorption coefficient, panels (a) and (c) and reduced scattering coefficient, panels (b) and (d) is reported. Panels (a) and (b) and panels (c) and (d) refer to epoxy-resin and silicone phantoms, respectively.

The precision of the three instruments, in terms of CV values, is presented in Fig. 6. Generally, precision is within 2% for all the three instruments for the epoxy-resin and the silicone phantoms. In particular, it is on the order of 0.5% to 1% in case of the epoxy-resin phantoms, and 1% to 1.5% in case of silicone phantoms, for absorption and reduced scattering coefficients. This indicates the good stability and robustness of the measurements with the three instruments in the whole spectral range considered. This also indicates that the phantom homogeneity is good, making it negligible for the effect of positioning during phantom measurements.

It is interesting to find that a similar behavior with respect to the spectral trend is revealed for the precision for the three instruments. To this extent, we mention that the precision for the absorption coefficient appears poorer around 690 and 880 nm for PHOOD and DOS systems for both the epoxy-resin and the silicone phantoms, as one can see in Figs. 6(a) and 6(c). In addition, we notice in Figs. 6(b) and 6(d) that the precision for reduced scattering coefficient worsens for larger wavelengths. This may be due to many complex factors, like photon count fluctuations, spectral characteristics of optical properties, and analysis employed. On the other hand, the precision of the absorption coefficient is affected less than that of the reduced scattering coefficient by the wavelength. Finally, for silicone phantoms, we can observe in Figs. 6(c) and 6(d) that precision of the absorption coefficient is overall slightly better than that of the reduced scattering coefficient for the three instruments.

### 3.3 Epoxy-Resin Phantoms

**Fig. 7:**
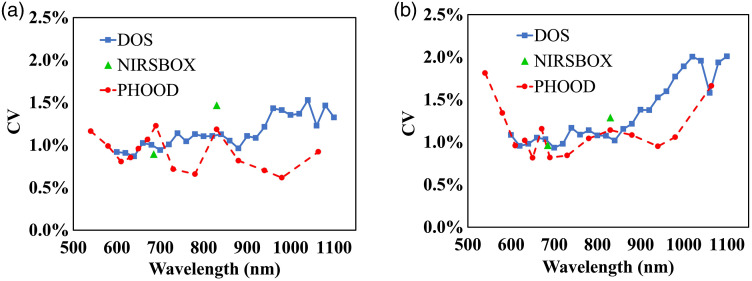
Intrabatch CV for the epoxy-resin phantoms, as a function of the wavelength, for absorption coefficient, panel (a) and reduced scattering coefficient, panel (b). Results obtained employing the three measurement systems are shown.

To study phantom differences within the same batch, the intrabatch CV of two epoxy-resin batches obtained by employing the three measurement systems is shown in Fig. 7. As shown in Fig. 7(a), the intrabatch CV for the absorption coefficient is within 1.5% for all the wavelengths employed, without any spectra-dependent consistency. As for the reduced scattering coefficient, the intrabatch CV is within 2% for all the wavelengths employed [see Fig. 7(b)]. In particular, for DOS and PHOOD systems, we observe an increase of intrabatch CV for wavelengths larger than 900 nm. Overall, intrabatch CV values for epoxy-resin phantoms are very close to the precision limit of the three instruments.

**Fig. 8:**
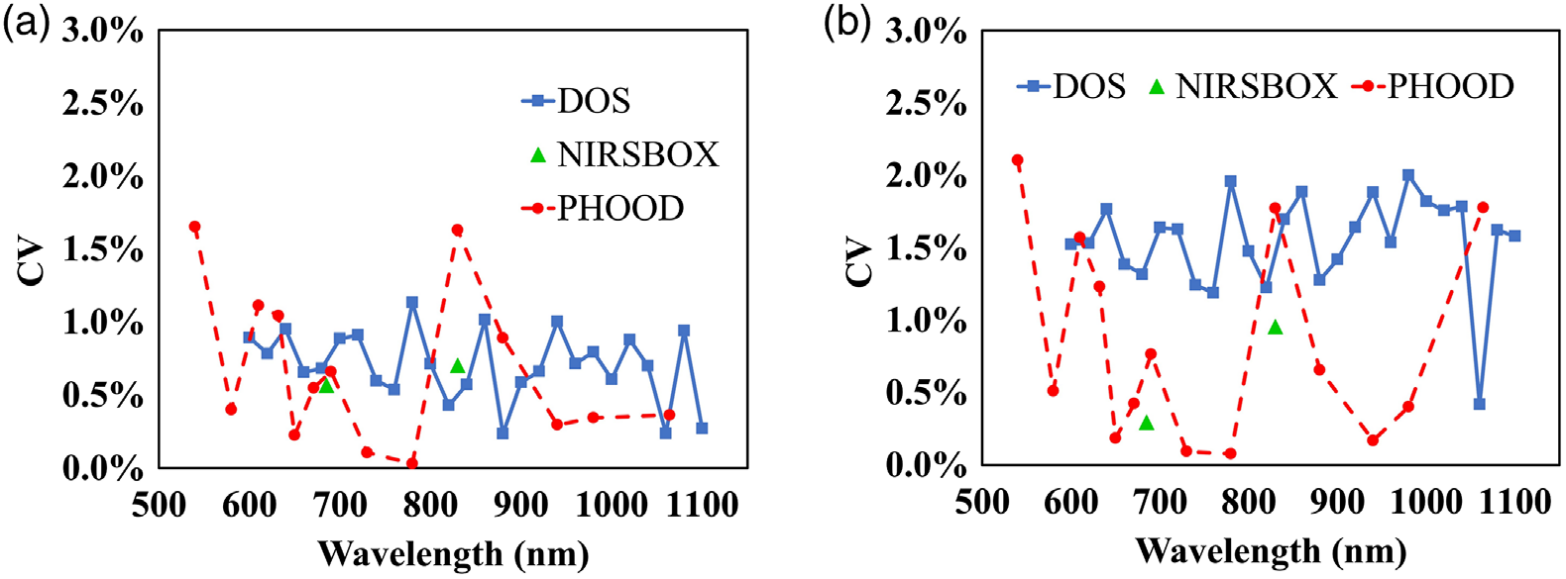
Interbatch CV for epoxy-resin phantoms, as a function of the wavelength, for absorption coefficient, panel (a) and reduced scattering coefficient, panel (b). Results obtained employing the three measurement systems are shown.

As for the differences between the two epoxy-resin batches, the interbatch CV is reported in Fig. 8 for the three measurement systems. The interbatch CV is approximately within 2% for absorption and reduced scattering coefficients, without apparent spectra-dependent consistency. In particular, the interbatch CV for the absorption coefficient seems lower than that for the reduced scattering coefficient on the whole with the three instruments. However, as we observe for the intrabatch CV, also for interbatch CV, we have values very close to the precision limit of the instrumentation. This denotes that the recipe for producing epoxy-resin phantom is reliable.

### 3.4 Silicone Phantoms

**Fig. 9:**
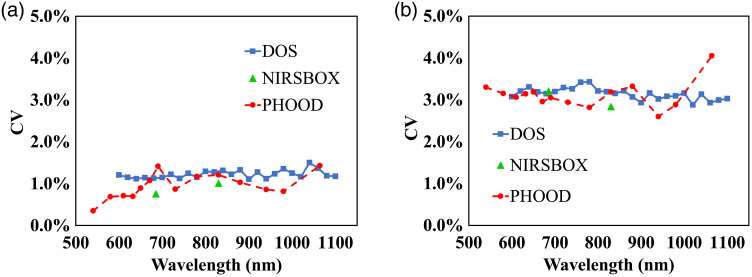
Intrabatch CV for silicone phantoms, as a function of the wavelength, for absorption coefficient, panel (a) and reduced scattering coefficient, panel (b). Results obtained employing the three measurement systems are shown.

The mean value of intrabatch CV for the two silicone batches is shown in Fig. 9 for the three measurement systems. As shown in Fig. 9, the intrabatch CV for silicone phantoms is about 1% for the absorption coefficient and 3% for the reduced scattering coefficient. Intrabatch CV for the three instruments is comparable, without any relevant spectral variations.

**Fig. 10:**
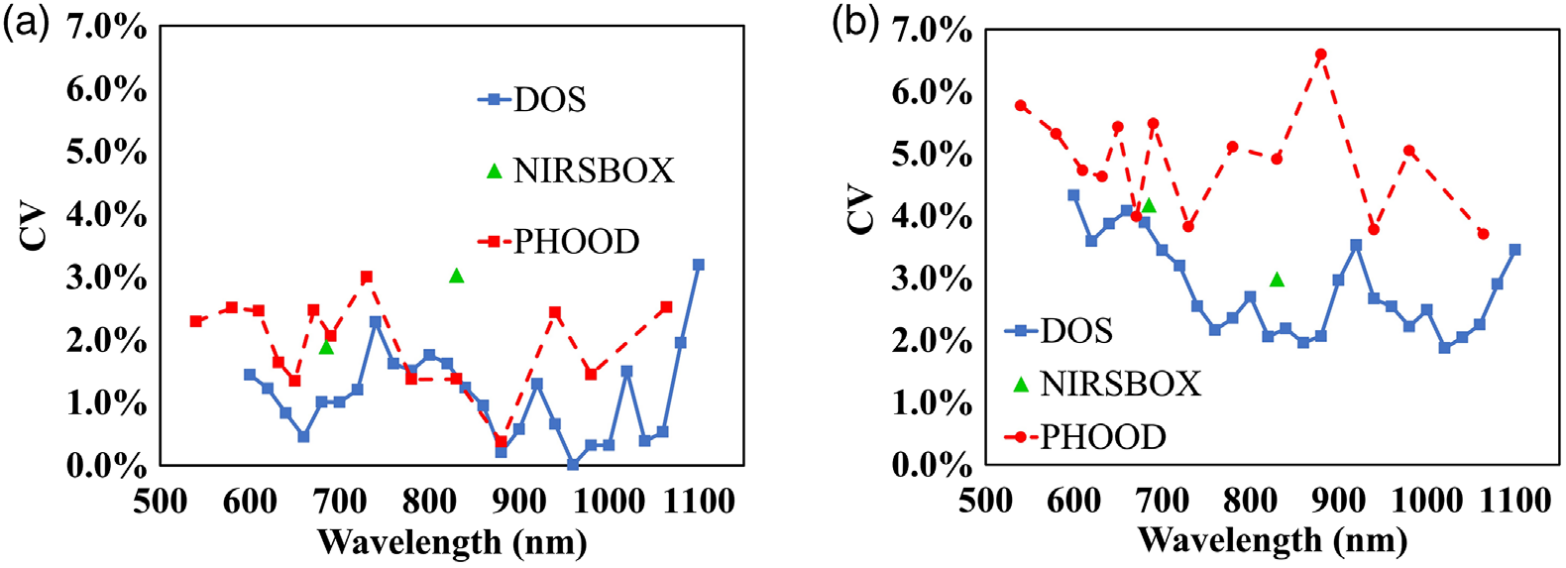
Interbatch CV for silicone phantoms, as a function of the wavelength, for absorption coefficient, panel (a) and reduced scattering coefficient, panel (b). Results obtained employing the three measurement systems are shown.

Interbatch CV for the two silicone batches is shown in Fig. 10 for the three measurement systems. The interbatch CV for the silicone is about 2% for the absorption and 5% for the reduced scattering spectra, on the whole a bit higher than their intrabatch CV. This is probably due to some critical steps during the silicone phantom preparation. As for the three instruments, DOS system witnesses lower interbatch CV for absorption and reduced scattering coefficients, being about 1% and approximately 3%, respectively. Probably, this is a consequence of the superior instrument precision shown by the DOS system. Again, no spectra-dependent consistency seems visible.

As for the two materials, the overall interbatch CV for silicone is higher, especially for the reduced scattering coefficient, compared with the interbatch CV for epoxy-resin shown in Fig. 8.

## 4 Conclusions

In this paper, we studied the reproducibility affecting homogeneous solid phantom fabrication for diffuse optics. As for fabrication materials epoxy-resin and silicone have been considered. Intra- and interbatch variability have been studied in terms of CV for the phantom optical parameters, which have been determined employing three different instruments for time-resolved diffuse spectroscopy that allowed spanning the wavelength range of 540 to 1100 nm.

The precision of the three instruments, determined considering repeated measurements on the same phantom, was similar, on the order of 0.5% to 1% in case of the epoxy-resin phantoms, and 1% to 1.5% in case of silicone phantoms, for absorption and reduced scattering coefficients.

As for epoxy resin phantoms, we obtained about 1% to 1.5% for intra- and interbatch CV. This value is very close to the precision limit of the instruments, and demonstrates a very good phantom reproducibility for the epoxy resin phantoms.

As for the silicone phantoms, we obtained about 1% and 3% as intrabatch CV for $\mu_{a}$ and $\mu_{s^{\prime }}$, respectively. On the other hand, we got a CV of about 2% and 3% to 5% when we considered the interbatch variability for $\mu_{a}$ and $\mu_{s^{\prime }}$.

Overall, the reproducibility results obtained with these two preparations for solid phantoms are very good. In particular, we can say that epoxy-resin phantoms can be assumed as “identical,” even if they come from different batches, resulting, then, well suited to be shared among research laboratories for intercomparison studies, distributed for multicenter clinical studies, or used in industrial standards. For the silicone phantoms we observed a larger variability, especially for the reduced scattering coefficient characterizing phantoms coming from different batches. This is probably due to some critical steps during their preparation. As we said before, the accurate estimation of optical properties is still an open issue; nevertheless, the opportunity to have solid phantoms with the same optical properties, even if not accurately known, is an indubitable advantage.

## Code, Data, and Materials Availability

Data used in this paper can be made available upon reasonable request to the authors.
